# Copy Number Variation Discovery in South African Nguni-Sired and Bonsmara-Sired Crossbred Cattle

**DOI:** 10.3390/ani13152513

**Published:** 2023-08-03

**Authors:** Bhaveni B. Kooverjee, Pranisha Soma, Magrieta A. van der Nest, Michiel M. Scholtz, Frederick W. C. Neser

**Affiliations:** 1 Animal Production, Agricultural Research Council, Pretoria 0062, South Africa; gscholtz@arc.agric.za; 2 Department of Biochemistry, Genetics and Microbiology, University of Pretoria, Pretoria 0028, South Africa; magriet.vandernest@fabi.up.ac.za; 3 Department of Animal Science, University of the Free State, Bloemfontein 9300, South Africa; neserfw@ufs.ac.za

**Keywords:** composite breed, crossbreeding, climate change, indigenous breeds, structural variation, panelcn.MOPS, whole genome sequencing, bovine genomics

## Abstract

**Simple Summary:**

Climate change plays a major role in livestock production. Hence the utilization of crossbreeding strategies allows for the improvement of animal production during harsh environmental conditions. The aim of this study was to identify the genetic differences in the F1 Nguni × Bonsmara and its reciprocal cross (Bonsmara × Nguni). This was achieved by studying the changes in structural variation, such as copy number variants in these two crosses. The major findings from this study have revealed several genes relating to adaption in these crossbred cattle.

**Abstract:**

Crossbreeding forms part of Climate-Smart beef production and is one of the strategies to mitigate the effects of climate change. Two Nguni-sired and three Bonsmara-sired crossbred animals underwent whole genome sequencing. Following quality control and file preparation, the sequence data were investigated for genome-wide copy number variation (CNV) using the panelcn.MOPS tool. A total of 355 CNVs were identified in the crossbreds, of which 274 were unique in Bonsmara-sired crossbreds and 81 unique in the Nguni-sired crossbreds. Genes that differed in copy number in both crossbreds included genes related to growth (SCRN2, LOC109572916) and fertility-related factors (RPS28, LOC1098562432, LOC109570037). Genes that were present only in the Bonsmara-sired crossbreds included genes relating to lipid metabolism (MAF1), olfaction (LOC109569114), body size (HES7), immunity (LOC10957335, LOC109877039) and disease (DMBT1). Genes that were present only in the Nguni-sired crossbreds included genes relating to ketosis (HMBOX1) and amino acid transport (LOC109572916). Results of this study indicate that Nguni and Bonsmara cattle can be utilized in crossbreeding programs as they may enhance the presence of economically important traits associated with both breeds. This will produce crossbred animals that are good meat producers, grow faster, have high fertility, strong immunity and a better chance of producing in South Africa’s harsh climate conditions. Ultimately, this study provides new genetic insights into the adaptability of Nguni and Bonsmara crossbred cattle.

## 1. Introduction

Copy number variation (CNV) is a type of structural variation that includes large-scale duplications, insertions and deletions which create imbalances in gene dosage and are described as the deletion or duplication of a genome copy number [1] that can have great functional and evolutionary impact [2]. For instance, CNVs play a key role in generating the required variation in a population [3]. Changes in gene copy number also influence gene dosage, unjustified gene fusion, disrupt gene functionality, and positional effects [4]. In addition, CNV can affect gene expression through duplication or deletion of gene regions, thereby affecting the overall phenotype expressed by the individual [5]. Overall, CNVs affect a wide range of phenotypic traits and animal performance.

CNVs were originally detected by approaches such as array-based Comparative Genomic Hybridization (aCGH), quantitative Polymerase Chain Reaction (qPCR), or using SNP arrays. Current studies involving CNV detection in cattle use SNP array technology [6,7], while a few studies have used the aCGH approach [4,8,9]. However, both approaches are limited by low accuracy of CNV location and CNV length estimation and are unable to detect CNVs along the entire genome sequence. For example, Hou et al. [10] utilized the Bovine HapMap SNP genotyping data to identify the genomic characteristics of cattle CNVs. They identified 682 candidate CNV regions where 56% of the CNVs overlap with cattle genes (1263) relating to immunity, lactation, reproduction and rumination. Another study by Wang et al. [7] characterized the CNVs across the South African Nguni genome using the Bovine SNP 50K Bead chip and next-generation sequencing technologies for CNV identification. The study found 458 genes located within 10 Mb of CNVRs, where 402 (87%) genes were unique to the Nguni population [7].

Studies have tried to improve the understanding of the underlying factors of the differences observed in crossbred cattle in their response to changing environmental conditions, such as heat stress [11,12,13]. For example, there is a crossbreeding program at the Vaalharts research station in the Northern Cape province of South Africa that studies the performance of different crossbred progeny [14]. One of the hybrids involved crossing purebred Nguni sires with Bonsmara dams and their reciprocal crosses. Observations indicated that in normal years, where no extreme temperatures, either too hot or cold, were observed, the Bonsmara-sired calves outperformed the Nguni-sired calves, but in hot and dry years, the Nguni-sired calves performed better in terms of body weight. In dry years BxN calves from the Nguni dams had much lower birth weights than NxB calves from the Bonsmara dams [14]. This may be due to Nguni’s adaptability to challenging climates. This may be an indication that calves from Nguni bulls were better adapted to harsh environments than calves from Bonsmara bulls [14].

Rapid advancement in next-generation sequencing (NGS) technology provides a more accurate approach to finding both common and rare CNVs, at a base-pair resolution [15]. Studies based on NGS have facilitated the discovery of smaller, previously unknown CNVs [16]. Thus, in this study, whole genome sequencing was utilized to identify CNVs with a higher effective resolution and increased sensitivity. Since CNVs are a significant source of genetic variability associated with phenotypic variation, the aim of this study was to survey genome-wide copy number differences between crossbred F1 progeny sired from either Nguni or Bonsmara sires crossed with Bonsmara and Nguni dams. Thus, the genomes of crossbred F1 progeny sired by either Nguni or Bonsmara bulls crossed with Bonsmara and Nguni dams were sequenced. Thereafter, the identification of genes that differ in copy number variants between the Nguni-sired and Bonsmara-sired calves was performed using a read-depth approach, panelcn.MOPS.

## 2. Materials and Methods

### 2.1. Animal Selection

Hair samples were collected from Nguni-sired (*n* = 2) and Bonsmara-sired (*n* = 3) male progeny and their respective parents originating from the Vaalharts Research station in the Northern Cape. Information regarding each crossbred animal, such as age and weight, is summarized in Supplementary Table S1. Parentage verification was performed using eleven ISAG-recommended microsatellite markers of each crossbred at the Animal Genetics Laboratory of the Agricultural Research Council—Animal Production campus. The number of animals used in this study for whole genome CNV analysis is similar to previous studies that utilized either one [17], two [18] or three [19,20] animals for genome-wide CNV detection in cattle.

### 2.2. DNA Extraction and Sequencing

The DNA was extracted from hair samples using the Qiagen DNeasy Blood and tissue kit as per the manufacturer’s protocol. The extracted DNA was run on a gel electrophoresis system to determine the integrity of the DNA. Briefly, 5 μL DNA was loaded in a 1% agarose gel and was run at 100 V for 30 min. Thereafter the gel was UV visualized with the Bio-Rad Molecular Imager Gel Doc™ XR + system. The DNA concentration was measured in ng/μL using the NanoDrop UV/Vis Spectrophotometer (NanoDrop ND-1000) and further verified using the Qubit 2.0 Fluorometer (Thermo Fisher Scientific, Waltham, MA, USA). All DNA samples were retained at a concentration of 50 ng/μL in preparation for NGS sequencing at the ARC-Biotechnology Platform. Sequence reads were filtered for base quality and adapter trimming using Trimmomatic v0.36 [21]. The trimming criteria used was that when four consecutive bases had an average Phred-like quality score of less than 33, the sequence read was trimmed. Subsequently, only pairs of DNA sequences with reads greater than 36 bp were retained for analysis. BWA-MEM v0.7.17 software [22] was used for sequence alignment to the reference genome (Bos_indicus_1.0 [23]). The *Bos indicus* reference (GCF_000247795.1_Bos_indicus_1.0) was chosen due to African Sanga cattle possessing unique indicine ancestry that is different from that found in modern cattle populations, such as the Brahman and Nellore [24]. The total number of reads, sequencing depths and mapping ratios are summarized in Supplementary Table S2. Each SAM file generated during the alignment process was converted to BAM (binary alignment map) files using the samtools “view” command and sorted using the samtools “sort” command and thereafter indexed using the samtools “index” command samtools [25]. Sequencing depth per individual was determined using the samtools “depth” command.

### 2.3. Copy Number Variation Analysis

Panelcn.MOPS is a bioinformatics tool used to identify copy number variation from whole genome sequencing data [26]. This tool is the modified build of Copy Number estimation by a Mixture of Poissons (cnMOPS) developed by Klambauer et al. [27]. Briefly, cnMOPS uses a Bayesian approach that comprises the use of a probabilistic model that explains the observed read counts by copy numbers based on the assumption that read counts in a region are distributed according to a mixture of Poisson’s [27]. The GUI version of the PanelcnMOPS program, CNV Detective (https://www.bioinf.jku.at/software/panelcnmops/, accessed on 3 November 2022), was used to detect copy number variations in the Nguni-sired and Bonsmara-sired crossbreds. Worthy to note initially, 3 NxB individuals and 3 BxN individuals were used for this analysis. However, one individual from the NxB group failed the first step of quality control and was removed. The remaining 3 BxN and 2 NxB individuals were used for further downstream analysis.

The input files for panelcn.MOPS includes the bam file for each individual to extract the read counts (RC). It also includes the bed file that is necessary to extract the regions of interest (ROI) that define the count windows. To create the bed files, the parameter “bamtobed” from bedtools was used [28]. Selecting the advanced option setting, parameters, such as the duplication threshold was fixed at 1.46, the deletion threshold was fixed at 0.57, along with minimum median RC/ROI ratio = 30 and the sex set as male. Quality control (QC) involves the calculation of the minimum median RC/ROI ratio, where samples with a median RC across all ROIs that is lower than 0.55 times the median of all samples fail the first step of the sample QC. Additionally, the ratio between the normalized RCs of each sample and the median across all remaining samples for each ROI is determined. When samples show a high variation in RC ratios, they fail the second QC step. CNVs labeled with ‘LowQual’ were regarded as non-significant and removed. All significant CNVs that passed quality control are reported in Supplementary Table S3. Gene annotation of the significant CNV regions was assessed based on the UMD3.1 bovine genome assembly using Ensembl (Ensembl Genes104).

## 3. Results

### 3.1. Copy Number Variants Summary Statistics

**Figure 1:**
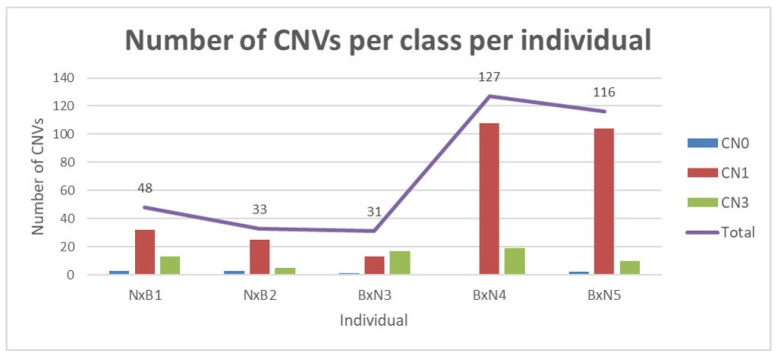
Graphical representation of the number of CNVs per class per crossbred individual, where CN0 = double copy deletion, CN1 = single copy deletion and CN3 = single copy duplication.

A total of 355 CNV regions were identified from the five crossbred animals. These CNV regions were comprised of 82% losses and 18% gains (Supplementary Figure S1). The highest number of deletion CNVs were detected on *Bos taurus* Autosome (BTA) 15 and the lowest on BTA27, while the highest number of duplication CNVs was detected on BTA 8 and no duplications were detected on BTA 1, 16, 27 and 28 (Supplementary Figure S2). Overall, the chromosomal distribution of CNV in the crossbreds demonstrates great variation in the copy number and total CNVs identified per chromosome (Supplementary Figure S2). These CNV regions cover 3.67 Mb of the Bos_indicus_1.0 genome assembly, which corresponds to ~0.15% of the bovine genomes. The estimated length of CNV regions varies from 230 bp to 274 Kb with an average of 9318 bp (Table 1). Individuals 1 (NxB1) and 2 (NxB2) from the Nguni-sired crossbreds and Individual 3 (BxN3), a Bonsmara-sired crossbred had CNVR ranging from 33–48. This was different from Individuals 4 (BxN4) and 5 (BxN5) of the Bonsmara-sired animals, which had three times more CNVR than individuals 1 (NxB1), 2 (NxB2) and 3 (BxN3) (Figure 1). The highest number of single-copy deletions was identified in individual BxN4 and the lowest in individual BxN3. The highest number of single duplications was detected in individual BxN4, while the lowest was found in individual NxB2.

### 3.2. CNV Gene Annotation

Genes were identified using Ensembl [29] to determine the potential functional roles associated with the identified CNVs. A total of 294 genes were identified across all individuals. However, genes that were present in two individuals per cross were reported. In the Bonsmara-sired crossbreds, 13 genes were identified (Table 2), while three genes were recognized in the Nguni-sired crossbreds (Table 3). The CNV genes detected play a role in multiple biological traits, such as fertility, growth, immunity, olfaction, lipid metabolism and disease.

## 4. Discussion

Panelcn.MOPs analysis allowed for the identification of genetic differences at the whole genome scale for two crossbred cattle genotypes (Nguni-sired and Bonsmara-sired). In this study, 355 CNV regions were identified in 5 individuals. Copy number variation analysis in various studies has shown great variation in the number of CNV regions in cattle [5,19,42]. A study by Wu et al. [43] identified 263 CNV regions in 792 Simmental cattle by means of PennCNV analyses, while Liu et al. [4] identified 177 CNV regions in 17 cattle breeds through whole-genome CGH array analysis. Zhan et al. [17] reported 520 CNV regions in a single Holstein Friesian bull using CNV-Seq, while Liu et al. [9] reported 490 CNV regions in 1092 Shanghai Holstein cattle using the cn.MOPS approach. It is worthwhile to note that the number of CNV regions detected in these studies varies dramatically. These discrepancies are attributed to sample size, the number of breeds used, the CNV detection approach and algorithms employed for identifying CNVs [44].

CNVs alter the expression of genes and change the phenotype of an individual as a result of deletions and duplications of genes in CNV regions. An interesting finding was that both crosses showed a similar trend of higher numbers of deletions than duplications, suggesting that there is less selection against duplication CNVs than deletions [45]. This trend was present across all samples and is in line with previous studies [20,42,46], which showed a similar trend of more CNV losses than gains in several breeds of indigenous Chinese cattle. However, two of the three Bonsmara-sired individuals (BxN 4 and 5) had almost thrice the number of deletions found in the Nguni-sired animals. Individual BxN3 had the lowest number of deletions of the Bonsmara-sired group, which may be a result of the difference in the relative humidity (RH) in the birth years ranging from 80.6% in 2017 (BxN 3) to 87.2% in 2019 (BxN 4 and 5) (South African Weather Services (SAWS), 2022). Moreover, the findings of Turner et al. [47] indicated that non-allelic homologous recombination (NAHR) is one of the major sources of CNVs, but it also showed that deletions occur at a higher rate than duplications in the male germline.

Results of this study suggested that the CNV regions between the crossbreds influence important phenotypic traits, as previously reported [2]. Several genes relating to cellular functioning and processing were identified in the crossbreds. For instance, in the Bonsmara-sired animals, ADP ribosylation factor like GTPase 6 interacting protein 4 (*AEL6IP4*) gene located on BTA17 was found to play a role in protein binding. In addition, the ubiquitin carboxyl-terminal hydrolase 17-like protein 6 (LOC109556616) located on BTA3 is responsible for the regulation of cellular processes [30], while in the Nguni-sired animals, the cationic amino acid transporter 3-like gene is responsible for amino acid transport [19]. Furthermore, cationic amino acids are crucial for the optimal growth of cattle and are controlled by cationic amino acid transporter activity [19]. Another gene identified in the Nguni-sired animals is the tumor necrosis factor receptor superfamily member 10A-like (LOC109563117) gene that plays a role in the apoptosis [48]. Furthermore, Higgins et al. [48] found a gene that is linked to the p53 signaling pathway in Charolaise cattle that were fed a high-concentrate diet. It is, therefore, possible that these genes will play an important part in the optimal functioning of the crossbreds, overall allowing crossbred animals to be better adapted to the environment.

Reproduction is one of the key factors driving the economic efficacy through sustainable meat production in the beef industry [49]. Therefore, utilizing breeds that have good reproductive performance will aid in the future growth of the cattle industry. In this study, genes associated with fertility-related factors were detected in the crossbred animals. In the BxN crossbreds, the ribosomal protein S28 (*RPS28*) gene, which plays a role in male fertility, is located on BTA7 and was identified in individuals BxN4 and BxN5. Sinha et al. [31] identified RPS28 as one of the candidate genes for bull fertility in Holstein Friesian cattle. A missense mutation T > C in the *RPS28* gene was shown to have a deleterious effect, which overall may affect the production of healthy and active spermatozoa [31]. In the NxB1 and NxB2 individuals, the disintegrin and metalloproteinase domain-containing protein 20-like (LOC109562432) gene located on BTA8 was previously found to play a role in male fertility [40]. The study by Maciel et al. [40] found the disintegrin and metalloproteinase domain-containing protein 20-like gene is involved with sperm capacitation, which is a process important for sperm viability before fertilization. The identification of these genes in the current study was expected, as only male individuals were included.

Growth is defined as an increase in size or weight, which is controlled by a large network of genes [50]. Other than the gene pool, the nutrients with which animals are supplied and the environment affect the growth of an animal. In this study, growth-related genes in the Bonsmara-sired crossbreds, such as secernin 2 (*SCRN2*) and *HES* family bHLH transcription factor 7 (HES7), both located on BTA19, were identified. *HES* family bHLH transcription factor 7 (HES7) is a transcriptional repressor involved in somitogenesis, which is responsible for vertebra and skeletal muscle development in mammals [34]. The role of *HES7* has not been studied in cattle. However, it was found to play a role in body size in sheep [35].

Immunity and disease-related genes were also identified in the Bonsmara-sired populations. For example, the SLA class II histocompatibility antigen, DQ haplotype D alpha chain (LOC109577039) gene located on BTA9 is known to play a role in the adaptive immunity [37]. This gene is part of the major histocompatibility complex that is present in all mammalian species and is important in the development of the immune system. Additionally, it is also an important candidate gene involved in susceptibility/resistance against various diseases [37]. Another gene, the class II major histocompatibility complex transactivator (*CIITA*) located on BTA25, is known to play a role in inflammatory response [38], as well as nematode resistance in Angus cattle [51]. The ‘deleted in malignant brain tumor 1’ (*DMBT1*) gene that is located on BTA26 was previously found to be associated with bovine TB in African Buffalo [52]. However, in cattle, the *DMBT1* was found to be involved with facial dysplasia syndrome in Holstein cattle [39]. A recent study revealed a CNV within the *DMBT1* gene to be present in two Chinese breeds that were also associated with growth, specifically body length [53]. An interesting finding was the high level of expression of the *DMBT1* gene in tuberculosis susceptible tissues [53].

Genes that play a role in the animal’s response to threats were identified in the Bonsmara-sired population. This includes genes that encode olfactory receptors (ORs) that help to alert animals of possible threats, such as predators. These receptors also assist animals in locating food and potential mates [54]. The OR genes are the largest gene family in the mammalian genome, and there are 881 OR genes in cattle [33]. In the Bonsmara-sired animals, the olfactory receptor 8J1-like gene was located on BTA15. In South Africa, predation of livestock was estimated to cost over ZAR1 billion in losses per year [55]. The sense of smell is important when animals are grazing on open and unprotected land, hence allowing them to perform efficiently in different grazing environments.

Overall, the genes detected in both crossbred populations provide insight into the possible mechanisms that may influence animal performance and response to environmental challenges.

## 5. Conclusions

This is the first study in South Africa to perform whole genome sequencing in Nguni and Bonsmara crossbred cattle. In addition, the genome-wide CNV analysis revealed 355 CNV regions in the crossbreds. Furthermore, this is also the first study to employ the panelcn.MOPS tool for CNV identification in cattle. The results of this study showed that copy number variations play a fundamental role in the Nguni and Bonsmara crossbred cattle, whereby different CNVs were identified in each cross. It should be noted that CNVs can affect gene networks and pathways due to changes in the gene copy number. CNVs are generated at a higher rate than point mutations in the genome, which can influence the evolution of genome complexity. Genes, such as fertility-related factors, were identified in both crossbreds. In the Bonsmara-sired crossbreds (calves from Nguni dams), genes relating to olfaction, lipid metabolism, growth, immunity and disease were detected, while genes relating to ketosis, amino acid transport and apoptosis were found in the Nguni-sired crossbreds (calves from Bonsmara dams). Overall, these results highlight the impact of parent selection on the genes inherited in each cross, which may further influence the adaption patterns of the F1 progeny. Additionally, adaptation patterns may help to identify genomic differences between the two sets of F1 progeny that can assist in explaining the differences observed in their ability to adapt to the warm, dry environment. Follow-up studies will include the parents and additional Bonsmara and Nguni crosses and should be expanded to include other crosses within the South African beef breeds to identify structural variation patterns in resulting F1 progeny.

## Figures and Tables

**Table 1:** Detailed information of CNVs detected in all crossbreds.

BTA No.	Chr Length (Mb)	Cumulative CNV Length	No. of CNVs	Mean Length (bp)	Median Length (bp)	Min Length (bp)	Max Length (bp)
1	161.11	1899	3	633	314	314	1271
2	140.68	12,375	7	1768	1201	746	4987
3	127.87	86,703	10	8670.3	2597	515	36,426
4	124.43	76,137	12	6345	3254	749	21,076
5	125.64	334,329	25	13,373	2858	935	96,975
6	122.65	52,759	11	4796	3236	1007	13,724
7	111.95	61,680	15	4112	2435	410	16,115
8	116.94	753,177	24	31,382	4920	230	110,871
9	108.1	25,279	7	3611	2357	896	9901
10	106.31	68,741	11	6249	959	428	56,532
11	110.26	263,313	17	15,489	2908	785	70,362
12	85.44	50,569	3	16,856	16,673	2637	31,259
13	84.43	32,268	10	3227	2570	1418	6174
14	81.41	8512	7	1216	1269	751	1464
15	84.8	380,029	37	10,271	962	929	274,172
16	77.91	12,954	5	2591	2583	1341	3674
17	76.52	25,833	9	2870	1682	1005	6364
18	65.95	231,509	26	8904	3256	575	101,156
19	65.32	246,360	27	9124	2473	296	164,086
20	75.86	86,586	3	28,862	18,014	7797	60,775
21	69.31	10,296	7	1471	1286	899	2081
22	61.89	48,558	12	4047	2478	837	11,298
23	53.33	188,807	15	12,587	1925	311	54,452
24	65.02	14,363	2	7182	7181.5	6451	7912
25	44.04	405,962	22	18,453	4102	968	153,354
26	51.86	118,236	3	39,412	58,321	1594	58,321
27	48.75	13,346	1	-	-	-	-
28	46.11	3465	3	1155	1231	374	1860
29	52.13	40,348	13	3104	1214	887	10,024
X	88.52	19,726	8	2466	1710.5	605	8804
Total	2634.54	3,674,119	355	9318	2478	230	274,172

**Table 2:** CNV genes identified in the Bonsmara-sired (BxN) crossbreds detected by panelcn.MOPS (RC > 0.55).

Sample	Function	BTA	CN	CNV Start	CNV End	Gene Start	Gene End	Gene	Full Name	References
BXN 3 + 4	GDP Binding	X	CN1	33981816	33982421	33975376	33985271	*RAB9B*	RAB9B, member RAS oncogene family	
BXN 4 + 5	Regulation of cellular processes	3	CN1	126931388	126932989	126930672	126933578	*LOC109556616*	ubiquitin carboxyl-terminal hydrolase 17-like protein 6	[30]
BXN 4 + 5	Fertility-related factors	7	CN1	15428095	15428909	15428030	15429198	*RPS28*	ribosomal protein S28	[31]
BXN 4 + 5		9	CN1	106343904	106344800	106342953	106345181	*LOC109563685*	probable plastid-lipid-associated protein 3, chloroplastic	
BXN 4 + 5	Lipid metabolism	14	CN1	564233	565697	563768	566833	*MAF1*	MAF1 homolog, negative regulator of RNA polymerase III	[32]
BXN 4 + 5	Olfaction	15	CN1	80006277	80007209	80006277	80007209	*LOC109569114*	olfactory receptor 8J1-like	[33]
BXN 4 + 5	Protein binding	17	CN1	55217115	55218797	55216878	55219698	*ARL6IP4*	ADP ribosylation factor like GTPase 6 interacting protein 4	
BXN 4 + 5	Body size	19	CN1	28307250	28309723	28306270	28309725	*HES7*	hes family bHLH transcription factor 7	[34,35]
BXN 4 + 5	Growth	19	CN1	39865977	39869076	39865484	39869235	*SCRN2*	secernin 2	[36]
BXN 4 + 5	Immunity	19	CN1	58635110	58637647	58635110	58637827	*LOC109573358*	CMRF35-like molecule 6	
BXN 4 + 5	Adaptive immunity	23	CN1	26144727	26199179	26144339	26199240	*LOC109577039*	SLA class II histocompatibility antigen, DQ haplotype D alpha chain	[37]
BXN 4 + 5	Inflammatory response	25	CN1	10600555	10645081	10600419	10646357	*CIITA*	class II major histocompatibility complex transactivator	[38]
BXN 4 + 5	Disease	26	CN1	43114715	43173036	43114701	43173429	*DMBT1*	deleted in malignant brain tumors 1	[39]

**Table 3:** CNV genes identified in the Nguni-sired (NxB) crossbreds detected by panelcn.MOPS (RC > 0.55).

Sample	Function	BTA	CN	CNV Start	CNV End	Gene Start	Gene End	Gene	Full Name	Reference
NxB 1 + 2	Fertility-related factors	8	CN3	7212767	7215055	7207894	7215055	*LOC109562432*	disintegrin and metalloproteinase domain-containing protein 20-like	[40]
NxB 1 + 2	Ketosis	8	CN0	9713354	9824225	9711258	9901658	*HMBOX1*	homeobox containing 1	[41]
NxB 1 + 2	Amino acid transport, Growth	18	CN1	61411652	61418513	61411629	61418610	*LOC109572916*	cationic amino acid transporter 3-like	[19]

## Data Availability

The raw data supporting the conclusions of this article will be made available by the authors upon reasonable request in line with ARC intellectual property regulations.

## Supplementary Materials

The following supporting information can be downloaded at: https://www.mdpi.com/article/10.3390/ani13152513/s1, Table S1: Information on the crossbred animals used in this study, Table S2: Sequencing depth and mapped reads of Nguni-sired and Bonsmara-sired crossbreds, Figure S1: CNV summary statistics of Copy number (CN) and number of CNVs for each CN class, Figure S2: Bar plot displaying the contribution of each copy number class to the total number of CNV calls per chromosome for all crossbred individuals, Table S3: All the CNVs detected in the crossbreds.
