# Supplementary material for: Copy Number Variation Discovery in South African Nguni-Sired and Bonsmara-Sired Crossbred Cattle
Source: Animals (Basel). 2023 Aug 3;13(15):2513. doi: 10.3390/ani13152513 (PMC10417447; doi:10.3390/ani13152513)
Supplement: Supplementary file 1 [file animals-13-02513-s001.zip › animals-2417528-supplementary.pdf]

Copy Number Variation Discovery in South African Nguni-Sired and Bonsmara-Sired Crossbred Cattle

Bhaveni B. Kooverjee, Pranisha Soma , Magrieta A. van der Nest, Michiel M. Scholtz and Frederick W. C. Neser

Table S1. Information on the crossbred animals used in this study.

Individual	SEX	Birth Weight (Kg)	Date of Birth
NxB1	Male	40	15-09-2019
NxB2	Male	38	18-09-2019
BxN3	Male	30	17-10-2017
BxN4	Male	40	18-09-2019
BxN5	Male	35	21-09-2019

Table S2. Sequencing depth and mapped reads of Nguni sired and Bonsmara sired crossbreds.

Individual	Total Reads	Mapped reads	Mapped (%)	Depth
NxB1	368,197,623	344,442,160	93.5	18.32
NxB2	335,282,240	314,851,328	93.9	15.91
BxN3	405,525,929	382,409,166	94.3	19.80
BxN4	294,132,129	275,079,007	93.5	14.74
BxN5	115,698,956	106,944,169	92.4	5.51

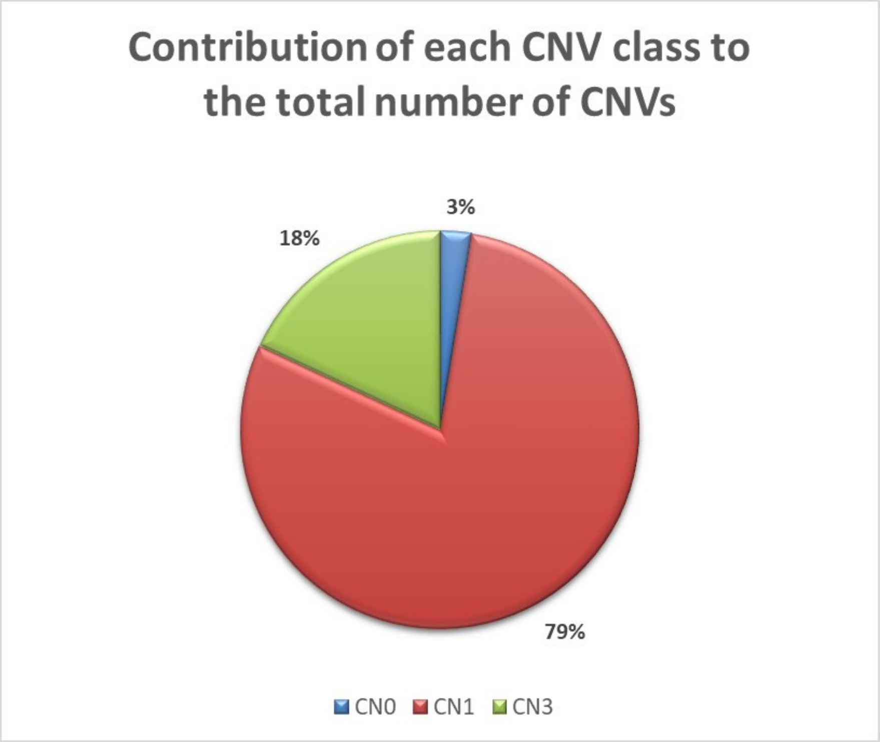

Figure S1. CNV summary statistics of Copy number (CN) and number of CNVs for each CN class. Where losses are represented by CN0 = double copy deletion, CN1 = single copy deletion and gains are represented by CN3 = single copy duplication.

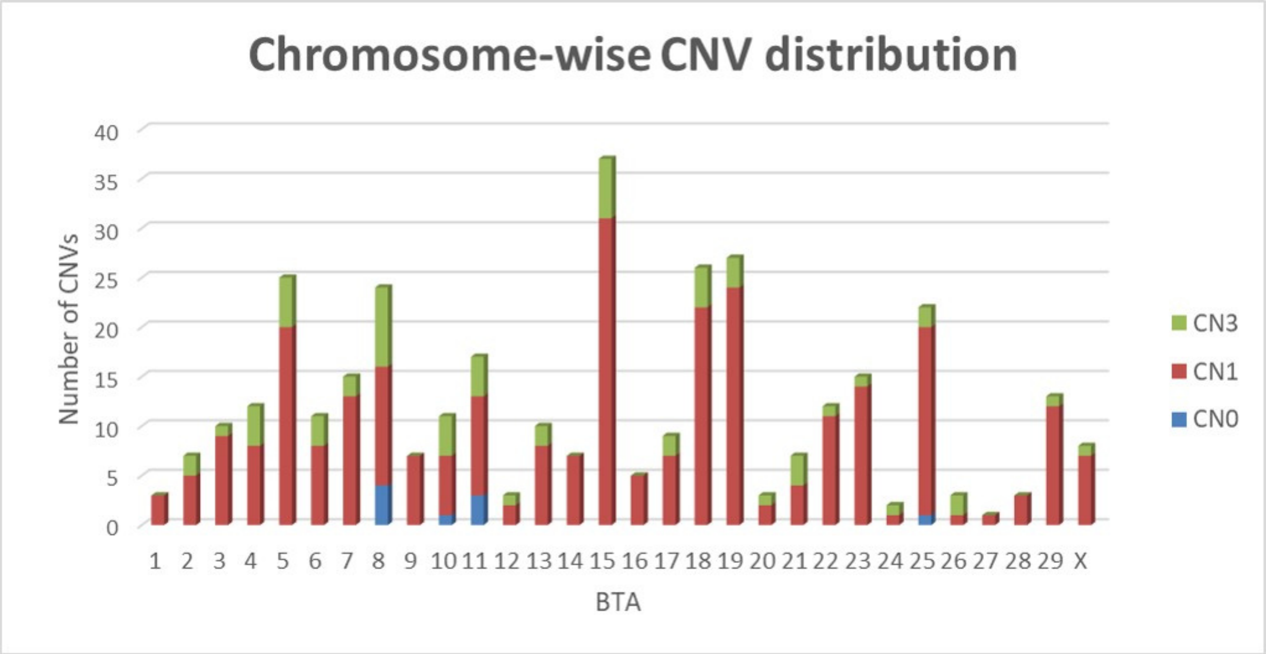

Figure S2. Bar plot displaying the contribution of each copy number class to the total number of CNV calls per chromosome for all crossbred individuals. CN0 (blue) and CN1 (red) represents the double and single copy number deletion events (losses), respectively. CN3 (green) represents the single duplication copy number events (gains).

Table S3. All the CNVs detected in the crossbreds.

Individ-ual	BTA	Gene	Gene Symbol	Gene Full Name	Start bp	End bp	RC	medRC	RC.norm	medRC.nor	RC.ratio	CN	Win-dow
BxN5	1	XP_019813263	FAM43A	family with sequence similarity 43 member A	74249656	74250927	27	144	54	94	0,57	CN1	-1
NxB1	1	XP_019820562	LOC109562066	keratin-associated protein 21-1-like	4007816	4008130	50	62.5	45	72	0.62	CN1	-1
BxN5	1	XP_019820600	LOC109562086	keratin-associated protein 21-1-like	4053048	4053362	9	50	18	33	0,55	CN1	-1
BxN5	2	XP_019824679	LOC109565286	ras-related GTP-binding protein C-like	5147265	5148466	24	124	48	82	0,59	CN1	-1
NxB1	2	XP_019833198	LOC109571278	40S ribosomal protein S6	115460800	115461594	67	108	60	95	0.63	CN1	-1
BxN5	2	XP_019840999	LOC109576808	gamma-crystallin C	101469299	101471209	75	164	151	105	1,44	CN3	-1
BxN4	2	XP_019825045	MSTN	myostatin	6524203	6529190	644	610.5	694	499	1.39	CN3	-1
BxN4	2	XP_019836041	SFN	stratifin	130454313	130455059	35	81	38	71	0.54	CN1	-1
BxN5	2	XP_019841229	TEX44	testis expressed 44	123376766	123377821	26	122	52	81	0,64	CN1	-1
BxN4	2	XP_019835341	TMEM200B	transmembrane protein 200B	128475257	128476939	63	120.5	68	98	0.69	CN1	-1
BxN4	3	XP_019810229	HAX1	HCLS1 associated protein X-1	17633636	17635983	114	211	123	185	0.66	CN1	-1
BxN4	3	XP_019812844	LOC109556172	tetratricopeptide repeat protein 9C-like	82387281	82387796	26	52	28	50	0.56	CN1	-1
BxN4	3	XP_019813083	LOC109556325	cytochrome P450 4A25-like	106107514	106121058	1604	1498.5	1729	1182	1.46	CN3	-1
NxB1	3	XP_019813083	LOC109556325	cytochrome P450 4A25-like	106107514	106121058	1393	1498.5	1245	1821	0.68	CN1	-1
NxB2	3	XP_019813083	LOC109556325	cytochrome P450 4A25-like	106107514	106121058	1224	1498.5	1141	1729	0.66	CN1	-1
BxN4	3	XP_019813637	LOC109556616	ubiquitin carboxyl-terminal hydrolase 17-like protein 6	126931388	126932989	1412	2795	1522	2386	0.64	CN1	-1
BxN5	3	XP_019813637	LOC109556616	ubiquitin carboxyl-terminal hydrolase 17-like protein 6	126931388	126932989	539	2779	1085	1702	0,64	CN1	-1
BxN5	3	XP_019844002	LOC109578994	T-cell surface glycoprotein CD1e, membrane-associated-like	12662087	12664934	74	334	149	222	0,67	CN1	-1
BxN5	3	XP_019813174	RNF220	ring finger protein 220	108232968	108269394	9535	42893	1920	2853	0,67	CN1	-1
BxN4	3	XP_019813906	S100A3	S100 calcium binding protein A3	18219995	18220729	40	81	43	70	0.61	CN1	-1
NxB1	4	XP_019814763	CLEC5A	C-type lectin domain containing 5A	108903617	108914151	1855	1484.5	1658	1139	1.46	CN3	-1
BxN4	4	XP_019815031	KLF14	Kruppel like factor 14	97691432	97692181	33	74	36	65	0.55	CN1	-1
NxB1	4	XP_019814378	LOC109557480	small nuclear ribonucleoprotein-associated protein B'-like	68630756	68632686	110	173.5	98	156	0.63	CN1	-1
BxN5	4	XP_019814597	LOC109557600	uncharacterized LOC109557600	95784146	95786909	135	294	272	200	1,36	CN3	-1
BxN4	4	XP_019814765	LOC109557707	maltase-glucoamylase, intestinal-like	108966455	108987531	1803	2980	1944	2935	0.66	CN1	-1
NxB1	4	XP_019814765	LOC109557707	maltase-glucoamylase, intestinal-like	108966455	108987531	3458	2980	3090	2171	1.42	CN3	-1
BxN4	4	XP_019814767	LOC109557710	cationic trypsin	109273876	109277621	171	349.5	184	310	0.59	CN1	-1
BxN5	4	XP_019815005	LOC109557911	metalloreductase STEAP1	76723661	76729984	19	106	38	71	0,54	CN1	-1
NxB2	4	XP_019815042	LOC109557960	olfactory receptor 9A4-like	108879330	108880274	107	182.5	100	162	0.62	CN1	-1
BxN4	4	XP_019815055	LOC109558007	olfactory receptor 6V1	110368624	110369565	59	126.5	64	108	0.59	CN1	-1
BxN4	4	XP_019815157	TAS2R38	taste 2 receptor member 38	108945807	108946814	93	166.5	100	166	0.6	CN1	-1
BxN4	4	XP_019814316	THAP5	THAP domain containing 5	61758291	61763340	610	567	658	489	1.35	CN3	-1
BxN5	5	XP_019815805	C5H12orf57	chromosome 5 C12orf57 homolog	10514133	10515573	31	157	62	104	0,6	CN1	-1
NxB1	5	XP_019815567	CCER1	coiled-coil glutamate rich protein 1	23549459	23550706	105	121	94	146	0.64	CN1	-1
NxB1	5	XP_019816120	DAZAP2	DAZ associated protein 2	31533772	31536630	439	324.5	392	279	1.41	CN3	-1
NxB1	5	XP_019816121	DAZAP2	DAZ associated protein 2	31533772	31536824	469	349	419	301	1.39	CN3	-1
NxB2	5	XP_019815966	HOXC6	homeobox C6	28862401	28863586	91	126.5	85	127	0.67	CN1	-1
BxN4	5	XP_019816528	INHBE	inhibin subunit beta E	60341758	60343051	67	143	72	122	0.59	CN1	-1
BxN4	5	XP_019817230	KCNA6	potassium voltage-gated channel subfamily A member 6	112031255	112032841	65	127.5	70	111	0.63	CN1	-1
NxB1	5	XP_019815332	LOC109558319	antigen WC1.1-like	10017212	10114187	12319	16287	1101	1707	0.64	CN1	-1
BxN5	5	XP_019815368	LOC109558369	olfactory receptor 8S1-like	34696160	34697095	56	110	113	79	1,43	CN3	-1
BxN5	5	XP_019815460	LOC109558521	uncharacterized LOC109558521	79619384	79654643	197	1054	397	668	0,59	CN1	-1
BxN5	5	XP_019815485	LOC109558561	phosphatidylinositol 4-phosphate 3-kinase C2 domain-containing subunit ga	98526588	98537798	39	204	79	136	0,58	CN1	-1
BxN3	5	XP_019815658	LOC109558771	olfactory receptor 6C74-like	63725804	63726742	117	162	101	154	0.66	CN1	-1
BxN5	5	XP_019815774	LOC109558889	antigen WC1.1-like	9780676	9841728	554	2857	1116	1730	0,65	CN1	-1
BxN4	5	XP_019816218	LOC109559139	tubulin alpha-1C chain	33594487	33601814	202	376.5	218	305	0.71	CN1	-1
BxN4	5	XP_019817113	LOC109559662	NKG2-A/NKG2-B type II integral membrane protein-like	106435686	106440244	1330	1198	1434	1057	1.36	CN3	-1
BxN3	5	XP_019817150	LOC109559684	C-type lectin domain family 2 member H-like	107400446	107479385	2756	1631.5	2390	1645	1.45	CN3	-1
BxN3	5	XP_019817476	LOC109559879	cytochrome P450 2D14	120030547	120034712	192	252	167	309	0.54	CN1	-1

BxN4	5	XP_019815531	MAFF	MAF bZIP transcription factor F	116835359	116836734	79	154.5	85	136	0.62	CN1	-1
BxN4	5	XP_019816487	METTL1	methyltransferase 1, tRNA methylguanosine	60004730	60007876	191	374	206	327	0.63	CN1	-1
BxN3	5	XP_019816802	MTERF2	mitochondrial transcription termination factor 2	75275935	75277092	136	157.5	118	170	0.69	CN1	-1
BxN4	5	XP_019815398	NXPH4	neurexophilin 4	60605357	60606554	50	119	54	105	0.51	CN1	-1
BxN4	5	XP_019816286	PCED1B	PC-esterase domain containing 1B	36235912	36237225	71	151.5	77	133	0.58	CN1	-1
BxN5	5	XP_019815562	PIANP	PILR alpha associated neural protein	10742532	10745390	55	272	111	173	0,64	CN1	-1
BxN4	5	XP_019815980	TARBP2	TARBP2 subunit of RISC loading complex	29475440	29480377	243	504.5	262	448	0.58	CN1	-1
BxN4	5	XP_019815981	TARBP2	TARBP2 subunit of RISC loading complex	29475440	29479767	226	428	244	375	0.65	CN1	-1
BxN4	6	XP_019817641	CYTL1	cytokine like 1	108904094	108908353	239	443.5	258	390	0.66	CN1	-1
BxN3	6	XP_019817826	FABP2	fatty acid binding protein 2	6817413	6819951	507	597	440	648	0.68	CN1	-1
BxN3	6	XP_019817736	HMX1	H6 family homeobox 1	120888846	120892751	405	243	351	244	1.44	CN3	-1
NxB1	6	XP_019817568	LOC109559966	rho GTPase-activating protein 20-like	5808805	5822529	7710	5194.5	6889	5516	1.25	CN3	-1
NxB1	6	XP_019817569	LOC109559967	rho GTPase-activating protein 20-like	5822994	5833052	3303	2318.5	2951	2302	1.28	CN3	-1
BxN3	6	XP_019817596	LOC109560002	mediator of RNA polymerase II transcription subunit 28-like	38069553	38076821	110	138	95	147	0.65	CN1	-1
BxN4	6	XP_019818050	LOC109560354	cytosol aminopeptidase-like	38065276	38067849	56	86	60	100	0.6	CN1	-1
NxB2	6	XP_019818388	LOC109560516	nucleosome assembly protein 1-like 1	86691522	86692697	110	170.5	103	162	0.64	CN1	-1
BxN4	6	XP_019818800	MFSD10	major facilitator superfamily domain containing 10	120079572	120082588	124	236	134	196	0.68	CN1	-1
BxN4	6	XP_019818823	TADA2B	transcriptional adaptor 2B	121678094	121679101	47	94.5	51	80	0.64	CN1	-1
BxN5	6	XP_019818658	ZNF518B	zinc finger protein 518B	109967525	109970761	15	81	30	53	0,57	CN1	-1
BxN5	7	XP_019820005	EBI3	Epstein-Barr virus induced 3	18446259	18452051	2474	11482	498	687	0,72	CN1	-1
BxN4	7	XP_019820558	EGR1	early growth response 1	49108075	49110394	150	283	162	229	0.71	CN1	-1
BxN5	7	XP_019818867	LOC109560819	uncharacterized LOC109560819	4894579	4910694	249	1232	501	811	0,62	CN1	-1
BxN4	7	XP_019819307	LOC109561359	histone H3.1	2503035	2503445	30	56	32	54	0.59	CN1	-1
BxN4	7	XP_019820074	LOC109561782	zinc finger protein 77-like	19580992	19588736	49	97.5	53	85	0.62	CN1	-1
NxB2	7	XP_019820636	LOC109562121	protocadherin alpha-10-like	51355150	51357585	313	244	292	209	1.4	CN3	-1
BxN5	7	XP_019819599	LYL1	LYL1 basic helix-loop-helix family member	10739244	10741251	41	198	83	132	0,63	CN1	-1
BxN5	7	XP_019819811	MCEMP1	mast cell expressed membrane protein 1	14972950	14975102	88	191	177	127	1,39	CN3	-1
BxN5	7	XP_019820419	MIDN	midnolin	42746072	42751923	108	497	217	331	0,66	CN1	-1
BxN4	7	XP_019819587	NACC1	nucleus accumbens associated 1	10710470	10713504	145	287.5	156	249	0.63	CN1	-1
BxN4	7	XP_019819760	PLPPR2	phospholipid phosphatase related 2	14214349	14218821	259	484	279	389	0.72	CN1	-1
BxN5	7	XP_019819844	RPS28	ribosomal protein S28	15428095	15428909	19	94	38	63	0,6	CN1	-1
BxN4	7	XP_019819055	SOWAHA	sosondowah ankyrin repeat domain family member A	44081575	44083470	85	190.5	92	169	0.54	CN1	-1
BxN4	7	XP_019819380	TMEM59L	transmembrane protein 59 like	4503872	4509183	299	571.5	322	476	0.68	CN1	-1
BxN5	7	XP_019819386	UBA52	ubiquitin A-52 residue ribosomal protein fusion product 1	4530808	4532137	35	175	70	114	0,61	CN1	-1
BxN5	8	XP_019822073	C8H9orf153	chromosome 8 C9orf153 homolog	83534817	83537090	54	260	109	173	0,63	CN1	-1
NxB2	8	XP_019822001	ENHO	energy homeostasis associated	80116078	80116308	27	47.5	25	44	0.57	CN1	-1
NxB1	8	XP_019821381	HMBOX1	homeobox containing 1	9713354	9824225	2E+05	208758	13595	19427	0.7	CN0	-1
NxB1	8	XP_019821384	HMBOX1	homeobox containing 1	9715044	9824225	2E+05	208593	13576	19411	0.7	CN0	-1
NxB2	8	XP_019821381	HMBOX1	homeobox containing 1	9713354	9824225	3E+05	208758	25505	19200	1.33	CN0	-1
NxB2	8	XP_019821384	HMBOX1	homeobox containing 1	9715044	9824225	3E+05	208593	25491	19185	1.33	CN0	-1
NxB1	8	XP_019821073	LOC109562432	disintegrin and metalloproteinase domain-containing protein 20-like	7212767	7215055	931	749	832	577	1.44	CN3	-1
NxB2	8	XP_019821073	LOC109562432	disintegrin and metalloproteinase domain-containing protein 20-like	7212767	7215055	819	749	764	548	1.39	CN3	-1
NxB2	8	XP_019821200	LOC109562612	grpE protein homolog 2, mitochondrial-like	1439169	1440208	68	106.5	63	101	0.62	CN1	-1
BxN4	8	XP_019821233	LOC109562645	interferon omega-1-like	24200072	24200659	37	73.5	40	64	0.62	CN1	-1
BxN4	8	XP_019821255	LOC109562668	interferon alpha-2-like	24509468	24510028	74	139	80	126	0.63	CN1	-1
BxN3	8	XP_019821258	LOC109562673	interferon alpha-3-like	24738447	24738920	60	72.5	52	88	0.59	CN1	-1
BxN4	8	XP_019821267	LOC109562689	GTP-binding nuclear protein Ran-like	57079946	57080599	49	89	53	91	0.58	CN1	-1
BxN4	8	XP_019821896	LOC109563117	tumor necrosis factor receptor superfamily member 10A-like	73381780	73438288	4889	4605	5271	4029	1.31	CN3	-1
BxN4	8	XP_019821892	LOC109563117	tumor necrosis factor receptor superfamily member 10A-like	73381860	73438288	4877	4582.5	5258	3998	1.32	CN3	-1

BxN4	8	XP_019821895	LOC109563117	tumor necrosis factor receptor superfamily member 10A-like	73383400	73438288	4783	4482	5157	3898	1.32	CN3	-1
NxB1	8	XP_019821894	LOC109563117	tumor necrosis factor receptor superfamily member 10A-like	73406726	73438288	2462	2832	2200	3590	0.61	CN1	-1
NxB2	8	XP_019821894	LOC109563117	tumor necrosis factor receptor superfamily member 10A-like	73406726	73438288	2502	2832	2333	3409	0.68	CN1	-1
BxN3	8	XP_019821902	LOC109563119	tumor necrosis factor receptor superfamily member 10B-like	73221637	73251777	4609	3226.5	3997	3106	1.29	CN3	-1
NxB1	8	XP_019821901	LOC109563119	tumor necrosis factor receptor superfamily member 10B-like	73221637	73228860	581	622	519	750	0.69	CN1	-1
NxB1	8	XP_019821902	LOC109563119	tumor necrosis factor receptor superfamily member 10B-like	73221637	73251777	2567	3226.5	2294	3739	0.61	CN1	-1
BxN3	8	XP_019821906	LOC109563126	uncharacterized LOC109563126	73877381	73878288	180	116	156	110	1.42	CN3	-1
BxN3	8	XP_019821910	LOC109563126	uncharacterized LOC109563126	73875671	73878288	245	149	212	147	1.44	CN3	-1
BxN4	8	XP_019821208	TMEM215	transmembrane protein 215	11423506	11424213	54	99	58	91	0.64	CN1	-1
BxN4	9	XP_019822476	LOC109563531	protein LEG1 homolog	67613842	67621192	450	850.5	485	752	0.64	CN1	-1
BxN5	9	XP_019822522	LOC109563604	mucin-5B-like	105112850	105115207	45	225	91	142	0,64	CN1	-1
BxN4	9	XP_019822571	LOC109563685	probable plastid-lipid-associated protein 3, chloroplastic [	106343904	106344800	37	80	40	70	0.57	CN1	-1
BxN5	9	XP_019822571	LOC109563685	probable plastid-lipid-associated protein 3, chloroplastic [	106343904	106344800	14	77	28	50	0,56	CN1	-1
BxN4	9	XP_019823118	LOC109564019	NKG2D ligand 3-like	87674881	87684782	985	2140.5	1062	1855	0.57	CN1	-1
BxN5	9	XP_019823143	LOC109564041	NKG2D ligand 4-like	90335088	90337723	250	1197	503	740	0,68	CN1	-1
BxN4	9	XP_019822733	TSPYL4	TSPY like 4	36168343	36169587	77	132.5	83	127	0.65	CN1	-1
BxN5	10	XP_019824197	ARF6	ADP ribosylation factor 6	42932396	42932923	12	64	24	43	0,56	CN1	-1
BxN5	10	XP_019823909	C10H14orf119	chromosome 10 C14orf119 homolog	22050282	22050710	20	97	40	65	0,62	CN1	-1
BxN5	10	XP_019824157	CAPN3	calpain 3	37624364	37680896	39392	191941	7933	11901	0,67	CN0	-1
BxN4	10	XP_019824789	ISCA2	iron-sulfur cluster assembly 2	88182226	88183504	81	151	87	145	0.6	CN1	-1
BxN3	10	XP_019823312	LOC109564212	histone H2B 1/2-like	22761651	22763417	141	160	122	186	0.66	CN1	-1
NxB2	10	XP_019823350	LOC109564304	olfactory receptor 4K13-like	26979380	26980318	87	117.5	81	125	0.65	CN1	-1
BxN4	10	XP_019823479	LOC109564486	olfactory receptor 11H4	26236942	26237886	71	137	77	121	0.64	CN1	-1
NxB1	10	XP_019823489	LOC109564499	olfactory receptor 4M1	26591155	26592114	168	109.5	150	110	1.36	CN3	-1
BxN4	10	XP_019823491	LOC109564501	olfactory receptor 4N5	26618628	26619554	190	185	205	153	1.34	CN3	-1
BxN5	10	XP_019823537	LOC109564562	olfactory receptor 4E1	24903574	24904587	57	115	115	79	1,46	CN3	-1
BxN4	10	XP_019824347	LOC109565103	protogenin-like	55620080	55623510	446	424	481	375	1.28	CN3	-1
BxN5	11	XP_019825118	CERCAM	cerebral endothelial cell adhesion molecule	102653730	102662859	118	604	238	394	0,6	CN1	-1
BxN3	11	XP_019826164	DNMT3A	DNA (cytosine-5) methyltransferase 3 alpha	76202981	76273343	8E+05	800100	71537	74050	0.97	CN0	-1
BxN5	11	XP_019826164	DNMT3A	DNA (cytosine-5) methyltransferase 3 alpha	76202981	76273343	2E+05	775296	36377	46938	0,78	CN0	-1
NxB2	11	XP_019826164	DNMT3A	DNA (cytosine-5) methyltransferase 3 alpha	76202981	76273343	8E+05	800100	78814	72766	1.08	CN0	-1
BxN4	11	XP_019825176	EGR4	early growth response 4	11617289	11619138	108	205.5	116	171	0.68	CN1	-1
BxN5	11	XP_019826672	ENTR1	endosome associated trafficking regulator 1	107850302	107853210	58	278	117	185	0,63	CN1	-1
BxN4	11	XP_019825581	GEMIN6	gem nuclear organelle associated protein 6	22337093	22340023	362	338	390	266	1.47	CN3	-1
BxN5	11	XP_019826351	GPR21	G protein-coupled receptor 21	97423868	97424917	23	112	46	74	0,62	CN1	-1
BxN4	11	XP_019825429	HTRA2	HtrA serine peptidase 2	10412204	10415095	319	295.5	344	231	1.49	CN3	-1
BxN5	11	XP_019825029	LOC109565543	neurexin-1-like	34836267	34837052	16	88	32	56	0,57	CN1	-1
BxN5	11	XP_019825086	LOC109565622	olfactory receptor 1J4-like	96636770	96637711	27	119	54	86	0,63	CN1	-1
BxN4	11	XP_019825089	LOC109565626	olfactory receptor 50-like	96749630	96750565	43	84.5	46	74	0.62	CN1	-1
BxN3	11	XP_019825151	LOC109565688	apical endosomal glycoprotein-like	109474934	109482420	924	566	801	601	1.33	CN3	-1
BxN4	11	XP_019825203	LOC109565727	zinc finger protein 2	2221118	2225630	316	581	341	503	0.68	CN1	-1
NxB2	11	XP_019826504	LOC109566513	long-chain fatty acid transport protein 4-like	102711159	102722911	96	190.5	90	170	0.53	CN1	-1
BxN4	11	XP_019826185	PFN4	profilin family member 4	77202115	77204722	345	323	372	265	1.4	CN3	-1
BxN4	11	XP_019825894	SH2D6	SH2 domain containing 6	51385146	51387599	127	256.5	137	226	0.61	CN1	-1
BxN5	12	XP_019827392	LOC109567086	multidrug resistance-associated protein 4-like	70533917	70565176	2348	12241	473	751	0,63	CN1	-1
NxB1	12	XP_019827286	MLNR	motilin receptor	18236311	18238948	168	231	150	226	0.66	CN1	-1
BxN5	12	XP_019827353	TMEM255B	transmembrane protein 255B	85045338	85062011	704	1306	1418	995	1,43	CN3	-1
BxN5	13	XP_019828244	BIRC7	baculoviral IAP repeat containing 7	55001360	55004660	58	287	117	191	0,61	CN1	-1
BxN5	13	XP_019828054	CTSA	cathepsin A	75435747	75441921	112	534	226	355	0,64	CN1	-1
NxB1	13	XP_019828023	DBNDD2	dysbindin domain containing 2	74514554	74516554	284	195.5	254	190	1.34	CN3	-1

BxN5	13	XP_019827598	LIME1	Lck interacting transmembrane adaptor 1	54711167	54712585	35	173	70	108	0,65		CN1	-1
BxN5	13	XP_019828216	LOC109567625	histone H2B type 2-E-like	43219513	43225045	36	193	72	125	0,58		CN1	-1
NxB2	13	XP_019828362	LOC109567781	uncharacterized LOC109567781	74056308	74058416	218	398	203	351		0.58	CN1	-1
NxB2	13	XP_019828506	LOC109567899	ankyrin repeat domain-containing protein 26-like	16741240	16746777	1706	1239.5	1591	1187		1.34	CN3	-1
BxN5	13	XP_019828833	LOC109568059	uncharacterized LOC109568059	42729753	42732785	62	300	125	182	0,69		CN1	-1
BxN4	13	XP_019828385	NKX2-2	NK2 homeobox 2	40884421	40886143	87	173	94	148		0.64	CN1	-1
NxB1	13	XP_019828600	OTUD1	OTU deubiquitinase 1	23824374	23825819	55	76	49	83		0.59	CN1	-1
BxN4	14	XP_019828992	FAM110B	family with sequence similarity 110 member B	24298166	24299245	40	79	43	66		0.65	CN1	-1
BxN4	14	XP_019829501	LOC109568570	uncharacterized LOC109568570	6542185	6543454	57	113.5	61	94		0.65	CN1	-1
BxN5	14	XP_019829248	LY6E	lymphocyte antigen 6 family member E	1387907	1388658	14	73	28	49	0,57		CN1	-1
BxN4	14	XP_019829224	MAF1	MAF1 homolog, negative regulator of RNA polymerase III	564233	565697	96	167.5	104	160		0.65	CN1	-1
BxN5	14	XP_019829224	MAF1	MAF1 homolog, negative regulator of RNA polymerase III	564233	565697	33	163	66	114	0,58		CN1	-1
BxN4	14	XP_019829592	RRS1	ribosome biogenesis regulator 1 homolog	30643062	30644159	60	119.5	65	104		0.62	CN1	-1
BxN4	14	XP_019829502	TRMT12	tRNA methyltransferase 12 homolog	15173091	15174479	91	167	98	154		0.64	CN1	-1
BxN5	15	XP_019830754	APOA5	apolipoprotein A5	25983416	25985345	45	210	91	140	0,65		CN1	-1
BxN5	15	XP_019830888	FCHSD2	FCH and double SH3 domains 2	52052204	52326376	20013	89784	4030	5972	0,67		CN1	-1
BxN3	15	XP_019831373	LIPT2	lipoyl(octanoyl) transferase 2	53580446	53581649	192	114	167	119		1.4	CN3	-1
BxN3	15	XP_019829769	LOC109568831	olfactory receptor 10A3-like	43878088	43880848	63	72	55	81		0.68	CN1	-1
BxN4	15	XP_019829775	LOC109568839	olfactory receptor 478-like	44101176	44102105	115	106	124	84		1.48	CN3	-1
BxN4	15	XP_019829798	LOC109568870	olfactory receptor 52E4	46524118	46525047	60	115	65	96		0.68	CN1	-1
BxN4	15	XP_019829811	LOC109568885	putative olfactory receptor 56B2	47127986	47128948	141	127	152	106		1.43	CN3	-1
NxB2	15	XP_019829866	LOC109568958	olfactory receptor 51H1-like	48985401	48986357	88	129.5	82	129		0.64	CN1	-1
BxN5	15	XP_019829894	LOC109568993	olfactory receptor 51A7-like	49696052	49706104	46	240	93	160	0,58		CN1	-1
BxN4	15	XP_019829971	LOC109569096	olfactory receptor 8J2-like	79715418	79716371	83	164	89	143		0.62	CN1	-1
BxN4	15	XP_019829974	LOC109569100	olfactory receptor 8K3-like	79752995	79760771	421	859.5	454	752		0.6	CN1	-1
BxN4	15	XP_019829979	LOC109569104	olfactory receptor 8K5-like	79852599	79861689	371	559	400	616		0.65	CN1	-1
BxN4	15	XP_019829984	LOC109569112	olfactory receptor 1044	79951410	79959360	112	163	121	190		0.64	CN1	-1
BxN4	15	XP_019829985	LOC109569114	olfactory receptor 8J1-like	80006277	80007209	46	69.5	50	87		0.57	CN1	-1
BxN5	15	XP_019829985	LOC109569114	olfactory receptor 8J1-like	80006277	80007209	19	46	38	62	0,61		CN1	-1
BxN4	15	XP_019829989	LOC109569117	olfactory receptor 8K5-like	80033013	80042102	428	711.5	461	681		0.68	CN1	-1
NxB1	15	XP_019829989	LOC109569117	olfactory receptor 8K5-like	80033013	80042102	803	711.5	718	503		1.43	CN3	-1
BxN3	15	XP_019829998	LOC109569131	olfactory receptor 5AL1-like	80345343	80346293	192	247	167	313		0.53	CN1	-1
BxN4	15	XP_019829998	LOC109569131	olfactory receptor 5AL1-like	80345343	80346293	138	247	149	278		0.54	CN1	-1
BxN4	15	XP_019830009	LOC109569143	olfactory receptor 5AK2-like	80886180	80887121	52	102.5	56	89		0.63	CN1	-1
BxN4	15	XP_019830062	LOC109569192	ecto-ADP-ribosyltransferase 5	51140201	51142085	100	210	108	181		0.6	CN1	-1
BxN4	15	XP_019830065	LOC109569192	ecto-ADP-ribosyltransferase 5	51140201	51142788	125	254.5	135	223		0.61	CN1	-1
BxN4	15	XP_019830112	LOC109569234	olfactory receptor 1440-like	83578635	83579579	41	76	44	76		0.58	CN1	-1
NxB2	15	XP_019830121	LOC109569244	olfactory receptor 2AT4-like	54106388	54107353	150	123.5	140	101		1.39	CN3	-1
BxN5	15	XP_019830139	LOC109569268	olfactory receptor 4X2-like	78496038	78496967	23	117	46	74	0,62		CN1	-1
BxN4	15	XP_019830195	LOC109569295	olfactory receptor 10A4	45233796	45234743	63	122	68	103		0.66	CN1	-1
NxB1	15	XP_019830206	LOC109569309	olfactory receptor 52N5-like	47034428	47035393	80	109.5	71	129		0.55	CN1	-1
BxN5	15	XP_019830214	LOC109569319	olfactory receptor 52E1-like	49814660	49824477	56	294	113	182	0,62		CN1	-1
BxN4	15	XP_019830216	LOC109569322	olfactory receptor 52D1	47728204	47729160	63	128.5	68	113		0.6	CN1	-1
BxN4	15	XP_019830292	LOC109569387	olfactory receptor 5G3-like	80559526	80560470	41	82	44	70		0.63	CN1	-1
NxB1	15	XP_019830355	LOC109569441	olfactory receptor 491-like	44134579	44135511	98	145	88	129		0.68	CN1	-1
NxB1	15	XP_019830862	LOC109569680	tripartite motif-containing protein 5-like	47459346	47467516	738	956.5	659	1062		0.62	CN1	-1
BxN5	15	XP_019831313	LOC109569947	olfactory receptor 9Q2-like	82024837	82025781	24	130	48	86	0,56		CN1	-1
NxB2	15	XP_019831319	LOC109569951	olfactory receptor 4B1-like	78580892	78581821	69	108.5	64	101		0.63	CN1	-1
NxB1	15	XP_019831321	LOC109569952	olfactory receptor 4X2-like	78550145	78551074	69	102	62	97		0.64	CN1	-1
BxN3	15	XP_019831414	LOC109570026	leupaxin	82720475	82721659	196	135	170	118		1.44	CN3	-1

NxB2	15	XP_019831422	LOC109570037	proteoglycan 3-like	81220712	81224171	721	823	672	992	0.68	CN1	-1
BxN4	16	XP_019832078	BTG2	BTG anti-proliferation factor 2	76581	78445	103	199.5	111	176	0.63	CN1	-1
BxN5	16	XP_019831750	MXRA8	matrix remodeling associated 8	48446584	48450076	72	351	145	232	0,62	CN1	-1
BxN5	16	XP_019831515	TMEM52	transmembrane protein 52	48105144	48106485	29	138	58	92	0,63	CN1	-1
BxN4	16	XP_019832356	TNFRSF25	TNF receptor superfamily member 25	44111342	44115016	212	390	229	331	0.69	CN1	-1
BxN5	16	XP_019832183	TNFRSF4	TNF receptor superfamily member 4	48557780	48560363	59	280	119	182	0,65	CN1	-1
BxN4	17	XP_019833471	ARL6IP4	ADP ribosylation factor like GTPase 6 interacting protein 4	55217115	55218797	99	189.5	107	159	0.67	CN1	-1
BxN5	17	XP_019833471	ARL6IP4	ADP ribosylation factor like GTPase 6 interacting protein 4	55217115	55218797	38	170	77	113	0,68	CN1	-1
NxB1	17	XP_019833364	B3GNT4	UDP-GlcNAc:betaGal beta-1,3-N-acetylglucosaminyltransferase 4	55854455	55855528	81	101	72	121	0.6	CN1	-1
BxN5	17	XP_019833158	CFAP73	cilia and flagella associated protein 73	64373703	64379615	252	557	507	370	1,37	CN3	-1
BxN4	17	XP_019832600	LOC109570913	6-pyruvoyl tetrahydrobiopterin synthase-like	56344185	56345190	141	133	152	106	1.43	CN3	-1
BxN4	17	XP_019832838	RHBDD3	rhomboid domain containing 3	72003310	72006140	129	272.5	139	241	0.58	CN1	-1
BxN5	17	XP_019832776	RPLP0	ribosomal protein lateral stalk subunit P0	65830937	65834585	88	431	177	274	0,65	CN1	-1
BxN4	17	XP_019832654	SUSD2	sushi domain containing 2	74668046	74674410	259	576	279	508	0.55	CN1	-1
BxN4	17	XP_019832750	ZNF891	zinc finger protein 891	46631368	46633005	129	251	139	216	0.64	CN1	-1
BxN3	18	XP_019833856	CA11	carbonic anhydrase 11	55101485	55107031	613	371.5	532	376	1.41	CN3	-1
NxB2	18	XP_019833996	CLEC11A	C-type lectin domain containing 11A	56740169	56742072	104	171.5	97	146	0.66	CN1	-1
BxN4	18	XP_019835304	DDX28	DEAD-box helicase 28	34503957	34505594	94	183	101	156	0.65	CN1	-1
BxN5	18	XP_019835375	IRF2BP1	interferon regulatory factor 2 binding protein 1	53187755	53189428	51	229	103	152	0,68	CN1	-1
BxN5	18	XP_019835622	KMT5C	lysine methyltransferase 5C	62527037	62531741	81	396	163	249	0,65	CN1	-1
BxN5	18	XP_019833801	LOC109571719	F-box only protein 27-like	48354921	48357943	138	280	278	192	1,45	CN3	-1
NxB1	18	XP_019833821	LOC109571739	carcinoembryonic antigen-related cell adhesion molecule 6-like	51153775	51195885	6308	7325	5637	7968	0.71	CN1	-1
NxB1	18	XP_019833877	LOC109571798	zinc finger protein 724-like	57249434	57257410	1484	2403.5	1326	2187	0.61	CN1	-1
BxN3	18	XP_019833912	LOC109571838	cationic amino acid transporter 3-like	61388529	61401929	2771	2252	2403	1655	1.45	CN3	-1
NxB1	18	XP_019833935	LOC109571861	leukocyte immunoglobulin-like receptor subfamily A member 6	62959168	62964097	281	353.5	251	376	0.67	CN1	-1
NxB1	18	XP_019833985	LOC109571908	zinc finger protein 420-like	57177877	57184068	1190	1818.5	1063	1667	0.64	CN1	-1
BxN5	18	XP_019834125	LOC109572041	leukocyte immunoglobulin-like receptor subfamily A member 6	63038725	63139881	1477	6223	2974	4139	0,72	CN1	-1
NxB2	18	XP_019835458	LOC109572793	cationic amino acid transporter 3-like	61057218	61058634	144	193	134	210	0.64	CN1	-1
NxB1	18	XP_019835644	LOC109572916	cationic amino acid transporter 3-like	61411652	61418513	1206	1379.5	1078	1748	0.62	CN1	-1
NxB2	18	XP_019835644	LOC109572916	cationic amino acid transporter 3-like	61411652	61418513	1067	1379.5	995	1660	0.6	CN1	-1
BxN4	18	XP_019835794	LOC109573033	carbohydrate sulfotransferase 6	2243625	2244812	52	104.5	56	92	0.61	CN1	-1
BxN5	18	XP_019835806	LOC109573039	transducin-like enhancer protein 4	38738501	38742016	74	347	149	229	0,65	CN1	-1
BxN5	18	XP_019834730	POLR2I	RNA polymerase II subunit I	46201941	46203132	26	137	52	83	0,63	CN1	-1
NxB1	18	XP_019833674	RGS9BP	regulator of G protein signaling 9 binding protein	42168835	42169548	63	90.5	56	88	0.64	CN1	-1
BxN5	18	XP_019835389	RRAD	RRAD, Ras related glycolysis inhibitor and calcium channel regulator	33646184	33648658	49	229	99	152	0,65	CN1	-1
BxN4	18	XP_019835823	SBK2	SH3 domain binding kinase family member 2	62384363	62388769	212	426	229	352	0.65	CN1	-1
BxN4	18	XP_019835825	SBK2	SH3 domain binding kinase family member 2	62385279	62388769	176	344	190	292	0.65	CN1	-1
BxN4	18	XP_019833725	UBE2M	ubiquitin conjugating enzyme E2 M	65909815	65912126	112	230	121	202	0.6	CN1	-1
BxN3	18	XP_019835202	ZNF524	zinc finger protein 524	62338136	62338933	138	81.5	120	82	1.46	CN3	-1
NxB2	18	XP_019833966	ZSCAN22	zinc finger and SCAN domain containing 22	65765398	65765973	58	67	54	82	0.66	CN1	-1
BxN5	18	XP_019833963	ZSCAN4	zinc finger and SCAN domain containing 4	65225457	65226922	33	146	66	97	0,68	CN1	-1
BxN3	19	XP_019836996	AKAP1	A-kinase anchoring protein 1	7171111	7182495	351	211	304	225	1.35	CN3	-1
BxN4	19	XP_019836425	CCDC182	coiled-coil domain containing 182	7789555	7790016	26	59	28	52	0.54	CN1	-1
BxN5	19	XP_019836514	CDK3	cyclin dependent kinase 3	57366146	57369178	57	291	115	184	0,62	CN1	-1
NxB1	19	XP_019838198	GJD3	gap junction protein delta 3	41869527	41870381	54	80.5	48	84	0.57	CN1	-1
BxN5	19	XP_019837051	GPS1	G protein pathway suppressor 1	52297747	52301611	61	279	123	186	0,66	CN1	-1
BxN4	19	XP_019836259	HES7	hes family bHLH transcription factor 7	28307250	28309723	108	232	116	205	0.57	CN1	-1
BxN5	19	XP_019836259	HES7	hes family bHLH transcription factor 7	28307250	28309723	40	222	81	147	0,55	CN1	-1
BxN4	19	XP_019837098	ICAM2	intercellular adhesion molecule 2	49766254	49780083	2383	2265	2569	1828	1.41	CN3	-1
BxN3	19	XP_019837981	KRT19	keratin 19	43089404	43093048	544	330	472	341	1.38	CN3	-1

BxN5	19	XP_019835983	LOC109573255	protein shisa-6 homolog	30535651	30699737	8786	39457	1769	2619	0,68	CN1	-1
BxN4	19	XP_019836080	LOC109573358	CMRF35-like molecule 6	58635110	58637647	150	277.5	162	285	0.57	CN1	-1
BxN5	19	XP_019836080	LOC109573358	CMRF35-like molecule 6	58635110	58637647	64	249	129	204	0,63	CN1	-1
NxB1	19	XP_019836348	LOC109573545	olfactory receptor 4D2-like	8191585	8192520	101	133.5	90	156	0.58	CN1	-1
BxN3	19	XP_019836351	LOC109573548	keratin-associated protein 3-1	42476426	42476722	33	45.5	29	51	0.57	CN1	-1
BxN5	19	XP_019837572	LOC109574185	C-C motif chemokine 14-like	13922731	13926942	97	464	195	309	0,63	CN1	-1
BxN4	19	XP_019837289	MRPL38	mitochondrial ribosomal protein L38	57442569	57446882	255	498.5	275	425	0.65	CN1	-1
BxN4	19	XP_019836963	ORMDL3	ORMDL sphingolipid biosynthesis regulator 3	41523157	41524726	118	209.5	127	198	0.64	CN1	-1
BxN5	19	XP_019837943	PPP1R1B	protein phosphatase 1 regulatory inhibitor subunit 1B	41265609	41273060	154	690	310	459	0,68	CN1	-1
BxN5	19	XP_019837863	RANGRF	RAN guanine nucleotide release factor	28447743	28448890	28	141	56	85	0,66	CN1	-1
BxN4	19	XP_019837109	SCRN2	secernin 2	39865977	39869076	176	328	190	289	0.66	CN1	-1
BxN5	19	XP_019837109	SCRN2	secernin 2	39865977	39869076	67	316	135	206	0,66	CN1	-1
BxN4	19	XP_019835972	SLC35G6	solute carrier family 35 member G6	27564204	27565790	90	166.5	97	140	0.69	CN1	-1
BxN4	19	XP_019836924	SRSF1	serine and arginine rich splicing factor 1	8031319	8033041	117	210.5	126	188	0.67	CN1	-1
BxN5	19	XP_019837431	TMEM95	transmembrane protein 95	27433322	27434741	32	156	64	104	0,62	CN1	-1
BxN5	19	XP_019837433	TMEM95	transmembrane protein 95	27433265	27434590	30	150	60	100	0,6	CN1	-1
BxN5	19	XP_019837430	TMEM95	transmembrane protein 95	27433322	27434498	27	134	54	89	0,61	CN1	-1
BxN5	19	XP_019836533	TNFSF13	TNF superfamily member 13	27795678	27797516	49	235	99	156	0,63	CN1	-1
BxN3	20	XP_019838522	LOC109574933	hydroxymethylglutaryl-CoA synthase, cytoplasmic	33442121	33449918	59	71	51	87	0.59	CN1	-1
BxN5	20	XP_019838542	LOC109574946	hydroxymethylglutaryl-CoA synthase, cytoplasmic-like	33465780	33483794	24	116	48	77	0,62	CN1	-1
BxN4	20	XP_019838442	RAI14	retinoic acid induced 14	41718533	41779308	7553	7340	8143	6049	1.35	CN3	-1
BxN5	21	XP_019839515	HDDC3	HD domain containing 3	21478756	21480017	29	142	58	94	0,62	CN1	-1
BxN5	21	XP_019839516	HDDC3	HD domain containing 3	21478756	21479940	28	135	56	90	0,62	CN1	-1
BxN5	21	XP_019838900	INSM2	INSM transcriptional repressor 2	46610927	46612591	29	157	58	99	0,59	CN1	-1
NxB1	21	XP_019839396	ISLR	immunoglobulin superfamily containing leucine rich repeat	34587991	34589277	153	113	137	94	1.46	CN3	-1
BxN3	21	XP_019839411	LOC109575609	myeloid-associated differentiation marker-like	19721269	19722168	179	108	155	108	1.44	CN3	-1
BxN5	21	XP_019839564	LOC109575719	duodenase-1-like	34881466	34883387	21	106	42	67	0,63	CN1	-1
BxN3	21	XP_019839597	LOC109575751	interferon alpha-inducible protein 27-like protein 2	59063083	59065164	229	137.5	199	143	1.39	CN3	-1
BxN4	22	XP_019840390	ABTB1	ankyrin repeat and BTB domain containing 1	61807382	61810559	145	288	156	244	0.64	CN1	-1
BxN4	22	XP_019840490	CCDC51	coiled-coil domain containing 51	52679199	52682107	195	358	210	322	0.65	CN1	-1
NxB1	22	XP_019839661	LOC109575847	uncharacterized LOC109575847	20116426	20117625	78	109	70	119	0.59	CN1	-1
BxN4	22	XP_019839762	LOC109575948	cathelicidin-5	52950138	52951988	80	154	86	131	0.66	CN1	-1
NxB2	22	XP_019839837	LOC109576004	IQ domain-containing protein F5	50047456	50048293	63	83.5	59	93	0.63	CN1	-1
BxN5	22	XP_019840383	LOC109576262	protein shisa-5	52713795	52715843	31	169	62	112	0,55	CN1	-1
BxN5	22	XP_019840574	LOC109576397	C-C chemokine receptor type 1	54633540	54634607	54	113	109	75	1,45	CN3	-1
BxN5	22	XP_019840596	LOC109576413	solute carrier family 26 member 6-like	52302230	52312701	32	167	64	101	0,63	CN1	-1
BxN5	22	XP_019840597	LOC109576413	solute carrier family 26 member 6-like	52301403	52312701	32	168	64	102	0,63	CN1	-1
BxN5	22	XP_019840598	LOC109576413	solute carrier family 26 member 6-like	52304357	52312701	30	158	60	96	0,62	CN1	-1
NxB2	22	XP_019839898	RPL29	ribosomal protein L29	49980095	49981722	133	203	124	203	0.61	CN1	-1
BxN5	22	XP_019839801	XIRP1	xin actin binding repeat containing 1	12657435	12661167	63	314	127	209	0,61	CN1	-1
NxB2	23	XP_019840902	LOC109576700	olfactory receptor 2G3-like	29378100	29379044	104	131.5	97	142	0.68	CN1	-1
NxB2	23	XP_019840925	LOC109576728	putative olfactory receptor 2W6	30859096	30860052	69	107	64	111	0.58	CN1	-1
BxN5	23	XP_019840953	LOC109576768	protein FAM177A1-like	29520416	29521051	10	58	20	39	0,51	CN1	-1
BxN3	23	XP_019840954	LOC109576769	interferon-induced transmembrane protein 3-like	27702658	27703095	390	236	338	238	1.42	CN3	-1
NxB1	23	XP_019841058	LOC109576861	butyrophilin subfamily 1 member A1-like	26451228	26458858	56	85.5	50	79	0.63	CN1	-1
BxN4	23	XP_019841114	LOC109576914	histone H4	31696818	31697129	26	55	28	48	0.58	CN1	-1
NxB1	23	XP_019841118	LOC109576917	histone H4	31964359	31964670	36	47.5	32	53	0.6	CN1	-1
NxB2	23	XP_019841177	LOC109576960	olfactory receptor 12D2-like	29187404	29188318	104	192.5	97	169	0.57	CN1	-1
BxN4	23	XP_019841310	LOC109577026	heat shock 70 kDa protein 1A	27266576	27268501	196	371.5	211	305	0.69	CN1	-1
BxN3	23	XP_019841328	LOC109577039	SLA class II histocompatibility antigen, DQ haplotype D alpha chain	26144727	26199179	672	787.5	583	903	0.65	CN1	-1

BxN4	23	XP_019841328	LOC109577039	SLA class II histocompatibility antigen, DQ haplotype D alpha chain	26144727	26199179	472	787.5	509	802		0.63	CN1	-1
BxN5	23	XP_019841328	LOC109577039	SLA class II histocompatibility antigen, DQ haplotype D alpha chain	26144727	26199179	174	672	350	572	0,61		CN1	-1
NxB1	23	XP_019841395	LOC109577084	MHC class I polypeptide-related sequence B-like	27768689	27772990	369	389.5	330	465		0.71	CN1	-1
NxB1	23	XP_019841397	LOC109577084	MHC class I polypeptide-related sequence B-like	27768689	27772849	367	384.5	328	456		0.72	CN1	-1
BxN4	23	XP_019841406	LOC109577088	BOLA class I histocompatibility antigen, alpha chain BL3-7	28611091	28614018	200	294	216	329		0.66	CN1	-1
BxN5	24	XP_019842014	LOC109577699	serpin B4-like	64737527	64745439	148	790	298	498	0,6		CN1	-1
BxN3	24	XP_019842037	LOC109577723	serpin B4-like	64487772	64494223	712	552.5	617	454		1.36	CN3	-1
BxN5	25	XP_019843323	AMDHD2	amidohydrolase domain containing 2	2527277	2532661	116	515	234	343	0,68		CN1	-1
BxN4	25	XP_019842595	ARHGDIG	Rho GDP dissociation inhibitor gamma	756307	760174	187	356	202	314		0.64	CN1	-1
BxN5	25	XP_019843324	ATP6V0C	ATPase H+ transporting V0 subunit c	2521811	2526515	97	460	195	306	0,64		CN1	-1
BxN4	25	XP_019843329	BRICD5	BRICHOS domain containing 5	2250272	2251704	84	167	91	144		0.63	CN1	-1
BxN4	25	XP_019843692	CIITA	class II major histocompatibility complex transactivator	10600555	10645081	3590	6140	3871	6146		0.63	CN1	-1
BxN4	25	XP_019843693	CIITA	class II major histocompatibility complex transactivator	10602114	10645081	3495	5944.5	3768	5948		0.63	CN1	-1
BxN5	25	XP_019843692	CIITA	class II major histocompatibility complex transactivator	10600555	10645081	1456	5688	2932	4385	0,67		CN1	-1
BxN5	25	XP_019843693	CIITA	class II major histocompatibility complex transactivator	10602114	10645081	1403	5510	2825	4243	0,67		CN1	-1
BxN5	25	XP_019843883	DCTPP1	dCTP pyrophosphatase 1	28400227	28403655	72	350	145	216	0,67		CN1	-1
BxN4	25	XP_019843607	HCFC1R1	host cell factor C1 regulator 1	2999407	3000478	70	134.5	75	114		0.66	CN1	-1
BxN5	25	XP_019843506	INO80E	INO80 complex subunit E	28055974	28060100	98	431	197	287	0,69		CN1	-1
BxN5	25	XP_019843507	INO80E	INO80 complex subunit E	28053584	28060100	145	645	292	429	0,68		CN1	-1
NxB1	25	XP_019842592	LOC109578041	liprin-alpha-1-like	268692	422046	4E+05	306194	36279	29398		1.23	CN0	-1
BxN5	25	XP_019842714	LOC109578155	olfactory receptor 7A17-like	29356523	29357491	34	170	68	113	0,6		CN1	-1
NxB1	25	XP_019843857	LOC109578789	F-box only protein 16-like	286973	314774	79259	59073	7082	5574		1.27	CN3	-1
BxN4	25	XP_019843955	LOC109578876	zymogen granule protein 16 homolog B-like	2770282	2772312	104	212	112	205		0.55	CN1	-1
BxN5	25	XP_019843689	METRN	meteorin, glial cell differentiation regulator	1065623	1067615	41	209	83	127	0,65		CN1	-1
BxN5	25	XP_019842597	NHLRC4	NHL repeat containing 4	948296	951131	56	272	113	175	0,65		CN1	-1
BxN4	25	XP_019843734	PKMYT1	protein kinase, membrane associated tyrosine/threonine 1	2937355	2941433	189	432.5	204	379		0.54	CN1	-1
BxN4	25	XP_019843428	PPP1R35	protein phosphatase 1 regulatory subunit 35	38189574	38190646	50	104	54	91		0.59	CN1	-1
BxN4	25	XP_019842596	PRR35	proline rich 35	946168	948155	162	154.5	175	125		1.4	CN3	-1
BxN5	25	XP_019842588	PRSS22	serine protease 22	2732522	2736853	97	440	195	293	0,67		CN1	-1
BxN5	26	XP_019808432	CUEDC2	CUE domain containing 2	23609262	23610856	36	177	72	126	0,57		CN1	-1
BxN4	26	XP_019808615	DMBT1	deleted in malignant brain tumors 1	43114715	43173036	8908	8477	9604	6837		1.4	CN3	-1
BxN5	26	XP_019808615	DMBT1	deleted in malignant brain tumors 1	43114715	43173036	3270	8056	6585	4877	1,35		CN3	-1
NxB2	27	XP_019809087	LOC109553490	butyrophilin-like protein 2	6409172	6422518	180	278.5	168	253		0.66	CN1	-1
BxN5	28	XP_019809575	COMTD1	catechol-O-methyltransferase domain containing 1	30374394	30376254	43	207	87	125	0,7		CN1	-1
BxN4	28	XP_019809845	FXYD4	FXYD domain containing ion transport regulator 4	12490639	12491870	62	137.5	67	121		0.55	CN1	-1
BxN4	28	XP_019809674	LOC109553863	pulmonary surfactant-associated protein D	35119694	35120068	20	42	22	40		0.55	CN1	-1
BxN5	29	XP_019810739	AP5B1	adaptor related protein complex 5 subunit beta 1	45870975	45873711	58	281	117	185	0,63		CN1	-1
BxN5	29	XP_019810392	GPR137	G protein-coupled receptor 137	44379581	44382203	51	257	103	160	0,64		CN1	-1
BxN4	29	XP_019809862	LOC109553987	upstream-binding factor 1-like protein 1	5521064	5521951	81	159.5	87	137		0.64	CN1	-1
BxN4	29	XP_019809865	LOC109553989	PRAME family member 8-like	5601743	5604477	1682	3356	1813	2920		0.62	CN1	-1
BxN4	29	XP_019809866	LOC109553991	upstream-binding factor 1-like protein 1	5644316	5645530	37	81.5	40	71		0.56	CN1	-1
BxN5	29	XP_019809868	LOC109553995	upstream-binding factor 1-like protein 1	5729657	5730871	31	142	62	94	0,66		CN1	-1
BxN4	29	XP_019809870	LOC109553996	tripartite motif-containing protein 64-like	5743698	5752631	1524	3089.5	1643	2675		0.61	CN1	-1
BxN4	29	XP_019809871	LOC109553997	tripartite motif-containing protein 43-like	5782921	5789192	1842	3528.5	1986	3030		0.66	CN1	-1
NxB1	29	XP_019809901	LOC109554033	putative olfactory receptor 8G3 pseudogene	28251977	28252912	30	48.5	27	45		0.6	CN1	-1
BxN5	29	XP_019809915	LOC109554053	putative olfactory receptor 8G3 pseudogene	28914465	28915388	26	125	52	79	0,66		CN1	-1
NxB1	29	XP_019810098	LOC109554217	olfactory receptor 8B3-like	28732247	28733173	153	223.5	137	208		0.66	CN1	-1
BxN5	29	XP_019810119	LOC109554241	olfactory receptor 8B3-like	29103319	29104248	27	133	54	88	0,61		CN1	-1
NxB1	29	XP_019810207	LOC109554300	tripartite motif-containing protein 64-like	5551374	5561398	1197	880.5	1070	809		1.32	CN3	-1
NxB2	X	XP_019811858	ARMCX1	armadillo repeat containing X-linked 1	30880957	30882318	51	73.5	48	75		0.64	CN1	-1

NxB2	X	XP_019810987	LOC109554802	PWWP domain-containing protein MUM1L1-like	22852889	22854949	259	374	241	357	0.68	CN1	-1
NxB2	X	XP_019811167	LOC109555062	nuclear RNA export factor 3-like	60822811	60831615	7532	6157	7023	5064	1.39	CN3	-1
NxB1	X	XP_019811560	LOC109555266	transcription elongation factor A N-terminal and central domain-containing	81170156	81171292	46	55	41	66	0.62	CN1	-1
BxN5	X	XP_019811929	LOC109555466	protein Shroom2-like	86251392	86254034	33	169	66	111	0,59	CN1	-1
BxN3	X	XP_019812026	RAB9B	RAB9B, member RAS oncogene family	33981816	33982421	41	50	36	62	0.58	CN1	-1
BxN4	X	XP_019812026	RAB9B	RAB9B, member RAS oncogene family	33981816	33982421	29	50	31	55	0.56	CN1	-1
BxN5	X	XP_019812004	SLITRK4	SLIT and NTRK like family member 4	15753773	15756286	26	132	52	88	0,59	CN1	-1
